# Bromodomain and extra-terminal domain (BET) proteins regulate melanocyte differentiation

**DOI:** 10.1186/s13072-020-00333-z

**Published:** 2020-03-10

**Authors:** Archit Trivedi, Aanchal Mehrotra, Caitlin E. Baum, Brandon Lewis, Tupa Basuroy, Thomas Blomquist, Robert Trumbly, Fabian V. Filipp, Vijayasaradhi Setaluri, Ivana L. de la Serna

**Affiliations:** 1 Department of Cancer Biology, University of Toledo College of Medicine and Life Sciences, 3035 Arlington Ave, Toledo, OH 43614 USA; 2 Present Address: Department of Genome Sciences, University of Washington School of Medicine, 1959 NE Pacific St, Seattle, WA 98195 USA; 3 Present Address: Cancer Center Division, Massachusetts General Hospital Harvard Medical School, 149 Thirteenth Street, 7th Floor, Charlestown, MA 02129 USA; 4 Department of Pathology, University of Toledo College of Medicine and Life Sciences, 3035 Arlington Ave, Toledo, OH 43614 USA; 5 Cancer Systems Biology, Institute of Computational Biology, Helmholtz Zentrum München, Ingolstädter Landstraße 1, München, 85764 Germany; 6 School of Life Sciences Weihenstephan, Technical University München, Maximus-von-Imhof-Forum 3, Freising, 85354 Germany; 7 Department of Dermatology, University of Wisconsin-Madison, The School of Medicine and Public Health, 1 S. Park Street, Madison, WI 53715 USA

**Keywords:** Bromodomain and extra-terminal domain, BET, JQ1, BRD4, MITF, ChIP-Seq, Epigenomics, Systems biology, Melanocyte differentiation, Melanoma, Pigmentation, Transcriptional networks

## Abstract

**Background:**

Pharmacologic inhibition of bromodomain and extra-terminal (BET) proteins is currently being explored as a new therapeutic approach in cancer. Some studies have also implicated BET proteins as regulators of cell identity and differentiation through their interactions with lineage-specific factors. However, the role of BET proteins has not yet been investigated in melanocyte differentiation. Melanocyte inducing transcription factor (MITF) is the master regulator of melanocyte differentiation, essential for pigmentation and melanocyte survival. In this study, we tested the hypothesis that BET proteins regulate melanocyte differentiation through interactions with MITF.

**Results:**

Here we show that chemical inhibition of BET proteins prevents differentiation of unpigmented melanoblasts into pigmented melanocytes and results in de-pigmentation of differentiated melanocytes. BET inhibition also slowed cell growth, without causing cell death, increasing the number of cells in G1. Transcriptional profiling revealed that BET inhibition resulted in decreased expression of pigment-specific genes, including many MITF targets. The expression of pigment-specific genes was also down-regulated in melanoma cells, but to a lesser extent. We found that RNAi depletion of the BET family members, bromodomain-containing protein 4 (BRD4) and bromodomain-containing protein 2 (BRD2) inhibited expression of two melanin synthesis enzymes, TYR and TYRP1. Both BRD4 and BRD2 were detected on melanocyte promoters surrounding MITF-binding sites, were associated with open chromatin structure, and promoted MITF binding to these sites. Furthermore, BRD4 and BRD2 physically interacted with MITF.

**Conclusion:**

These findings indicate a requirement for BET proteins in the regulation of pigmentation and melanocyte differentiation. We identified changes in pigmentation specific gene expression that occur upon BET inhibition in melanoblasts, melanocytes, and melanoma cells.

## Introduction

The bromodomain and extra-terminal (BET) family of proteins contains BRDT, BRD2, BRD3, and BRD4 [1]. BRD2, BRD3, and BRD4 are ubiquitously expressed while BRDT is expressed mainly in testes. BET proteins have two bromodomains, defined as motifs of 110 amino acids that recognize acetylated lysines, as well as a conserved extra-terminal domain which mediates additional protein–protein interactions. BET family members play important roles in transcription by binding acetylated histones and interacting with components of the transcriptional machinery and chromatin remodeling enzymes. They can have overlapping, distinct, and even opposing roles in transcription depending on the promoter and pathway [2–4]. BRD2 and BRD4 have been shown to interact with the mediator complex while BRD4 can also interact with P-TEFb to promote transcription [5–8]. BRD4 contributes to the regulation of nucleosome and higher order chromatin structure [9, 10] but by a different mechanism than does BRD2, which plays a role unique from BRD4 by interacting with CTCF [11].

In recent years, BET proteins have emerged as drivers of tumorigenesis in diverse human cancers. Translocations that generate oncogenic BRD4 or BRD3 fusion proteins have been identified in NUT-midline carcinoma [12, 13]. In other malignancies, BRD4 associates with cancer-specific super enhancers, hematopoietic transcription factors in acute myeloid leukemia, YAP/TAZ, and other pro-tumorigenic transcription factors to promote proliferation and cancer progression [14, 15]. The discovery of the first BET inhibitor, JQ1 made it possible to target BET proteins as a therapeutic strategy against cancer [16]. JQ1 is a stereo-specific, cell-permeable small molecule that binds competitively to acetyl-lysine recognition motifs present in BET proteins which removes them from chromatin and disrupts their tumorigenic functions [14, 17, 18]. Clinical and pre-clinical studies using JQ1 and next generation BET inhibitors are in progress for hematological cancers, and solid tumors including NUT-midline carcinoma and melanoma [19].

Although recent studies have elucidated BET proteins as potential therapeutic targets in melanoma, their function in normal melanocytes is not known. The melanocyte lineage arises from neural crest cell derived melanoblasts which differentiate into pigmented melanocytes and give skin and hair their characteristic color [20]. The pigment, melanin, is synthesized in organelles called melanosomes and then transferred to surrounding keratinocytes in a process known as melanogenesis [21]. This involves a series of chemical reactions catalyzed by tyrosinase (TYR) and tyrosinase related proteins (TYRP1). Additional proteins regulate melanosome structure, function, and distribution. The level of pigmentation is determined by the amount and type of melanin, as well as the size, number, and distribution of melanosomes. These are all determined by genetic factors and modulated by physiological and environmental conditions. The master regulator of melanocyte development, melanocyte inducing transcription factor (MITF, historically also called microphthalmia-associated transcription factor) coordinates with other melanocyte-specific transcription factors to regulate melanogenesis in normal melanocytes and play critical roles in melanoma [22]. An increasing number of studies show that epigenetic regulators are also required for regulation of both normal melanocyte function and melanoma tumorigenicity [23]. Furthermore, inherent features of the melanocyte differentiation program contribute to melanoma progression and resistance to therapeutics [24, 25]. Therefore, a better understanding of the function of BET proteins in melanocyte differentiation may provide additional insight into their function in melanoma.

In this study, we tested the hypothesis that BET proteins regulate melanocyte differentiation by interacting with MITF. We treated melanoblasts with the BET inhibitor, JQ1 and found that JQ1 prevented melanoblasts from differentiating into pigmented melanocytes. BET inhibition decreased expression of pigment-specific genes and slowed proliferation. Our experiments also revealed that both BRD4 and BRD2 interact with MITF and contribute to the regulation of melanocyte-specific gene expression.

## Materials and methods

### Cell culture

Melb-a cells (melanoblasts) were maintained in growth medium (RPMI, 10% fetal calf serum, 40 pM fibroblast growth factor and 10 ng/ml stem cell factor) and differentiated in differentiation medium [DMEM, 10% fetal calf serum, 2 nM alpha melanocyte stimulating hormone (NDP-alpha MSH) and 200 nM phorbol-myristate-acetate] as previously described [26]. Neonatal human epidermal melanocytes (NHEMs) were isolated and cultured as previously described [27]. Human embryonic kidney (HEK) 293T cells were obtained from ATCC. They were cultured in DMEM Media (Life Technologies, Grand Island, NY, USA) containing 10% fetal bovine serum. The pair of active (+) [(*S*)-enantiomer] and inactive (−) [(*R*)-enantiomer] JQ1 stereoisomers were purchased from BPS Biosciences (San Diego, CA, USA) and each dissolved in DMSO.

### Cell counts

Cells were trypsinized and resuspended in media at the indicated times. Cell counts were taken using the Scepter 2.0 handheld automated cell counter (Millipore-Sigma, Burlington, MA, USA).

### Cell cycle analysis

Approximately 1 × 10^6^ cells were fixed with 100% ethanol for 1 h, stained with a propidium iodide (PI)-RNAse solution (Millipore-Sigma, Burlington, MA, USA) for 30 min and loaded on a FACS-Calibur (BD Biosciences, Franklin Lakes, NJ, USA) at the University of Toledo Flow Cytometry Core Facility. Data were analyzed using Cell Quest Pro (BD Biosciences).

### Apoptosis assay

Cells were stained with the Guava Nexin Annexin V reagent (Millipore-Sigma) as we previously described and analyzed on a FACS-Calibur (BD Biosciences) at the University of Toledo Flow Cytometry Core Facility [27].

### Melanin assay

The melanin assay was performed as previously described [26]. Cells were counted and lysed in 0.1 M NaOH for 30 min. Absorbance was read using a spectrophotometer (Molecular Devices) at 475 nM and melanin content was calculated based on a standard curve using synthetic melanin. Melanin content was then normalized to cell number.

### RNA-seq

Total RNA was prepared from vehicle (DMSO) or (+)JQ1 treated Melb-a melanoblasts by Trizol extraction (Invitrogen, Carlsbad, CA, USA) as previously described [26]. All experiments were performed in triplicate. Library preparation, quality assessment, and sequencing were performed by Bio Basics Inc. (Markham, Ontario, Canada). mRNA was purified from total RNA using poly-T oligo-attached magnetic beads. First strand cDNA was synthesized using random hexamers and M-MuLV Reverse Transcriptase. Library fragments were purified and PCR amplified. Paired-end sequencing was performed on Illumina HiSeq 4000. The raw reads were filtered by removing reads containing *N* > 10% (where *N* represents bases that could not be determined). The *Q* score (quality value) of over 50% bases of the read was less than or equal to 5.

### Bioinformatics

Raw RNA-seq data were converted to sequence reads using CASAVA. Raw reads were filtered to remove low-quality reads and reads with adaptors. The clean reads were mapped to the Ensembl mouse genome (grcm38/mm10) using Tophat2 (v2.0.9) [28]. Differential gene expression between vehicle and (+)JQ1-treated samples was determined using the DESeq2 package 2.1.6.3 [29]. The resulting *p* values were adjusted using Benjamini and Hochberg’s approach for controlling false discovery rate (FDR) and set at *p* < 0.05. Gene ontology (GO) analysis was performed using the GOseq R package [30]. In addition, differential gene expression was analyzed with Kegg pathway analysis [31]. Comparative analyses with previously published data sets were performed with the CLC Genomic Workbench (Qiagen, Hilden, Germany). For this comparative analysis, mouse and human gene homologues were matched using HOM Mouse Human Sequence.rpt (accessed from Jackson laboratories, http://www.informatics.jax.org; accessed 03/29/2019), two tab delimited files of Ensembl homog IDs and gene names (human and mouse, accessed from grch37.ensembl.org/biomart; accessed 03/29/2019), and when necessary, manual matching of homologues using the online directory GeneCards.

Published ChIP-seq datasets in GEO and Encode in bigWig format were visualized directly as Custom Tracks on the UCSC Genome Browser. To determine the number of overlapping peaks for MITF and BRD4, the GSM2527370 dataset was used for MITF, and the GSM2700494 dataset was used for BRD4. The BED files were downloaded from the Cistrome database: http://cistrome.org/db/#/ and imported into RStudio as GRanges. The overlapping peaks were identified using the peakPermTest function in the ChIPpeakAnno package available through Bioconductor. The peakPermTest function was used to perform a permutation test in order to determine the statistical significance of the overlap.

### RNA isolation and quantitative real-time PCR

Total RNA was reverse transcribed into cDNA using the Quantitect Reverse Transcription kit (Qiagen). Quantitative PCR (qPCR) was performed in SYBR Green master mix (Qiagen) with an Applied Biosystems 7500 PCR and analyzed with the SDS software as described [32]. Mouse *Tyr*, *Tyrp1*, *Trpm1*, *Brd2*, and *Brd4* mRNA levels were normalized to mouse *Rpl7*. Human *TYRP1* mRNA levels were normalized to human *RPL19*. All primers were obtained from Integrated DNA Technologies (IDT) (Coralville, IA, USA). Primer sequences for murine genes were: *Tyr*: forward: 5′-TTC AAA GGG GTG GAT GAC CG-3′ and reverse: 5′-GAC ACA TAG TAA TGC ATC C-3′, *Tyrp1*: forward: 5′-GCC CCA ACT CTG TCT TTT CTC AAT-3′ and reverse: 5′-GAT CGG CGT TAT ACC TCC TTA GC-3′, *Trpm1*: forward: 5′-CCT ACG ACA CCA AGC CAG AT-3′ and reverse: 5′-GAC GAC ACC AGT GCT CAC AC-3′, *Brd2*: forward: 5′-GCCCTTCTATAAGCCAGTGG-3′ and reverse: 5′-AAACTCCTGTGCATCCCG-3′, *Brd4*: forward: 5′-TAA AAA CTC CAA CCC CGA TG-3′ and reverse: 5′-TGC TCT CCG ACT CAG AGG AT-3′, *Rrpl7*: forward: 5′-GGA GGA AGC TCA TCT ATG AGA AGG-3′ and reverse: 5′-AAG ATC TGT GGA AGA GGA AGG AGC-3′. Primer sequences for human genes were: *TYRP1*: forward: 5′-TGG GAT CCA GAA GCA ACT TT-3′ and reverse: 5′-TGT GGT TCA GGA AGA CGT TG-3′, RPL19: forward: 5′-AAA CAA GCG GAT TCT CAT GG-3′ and reverse: 5′-TTG GTC TCT TCC TCC TTG GAT-3′.

### Antibodies

The MITF (ab12039) and BRD4 (ab128874) antibodies were from Abcam (Cambridge, MA, USA). The BRD2 antibody (A302-583A-M) was from Bethyl (Montgomery County, Texas, USA). The BRD3 (sc-81202), TYR (sc-7833), and TYRP1 (sc-10443) antibodies were from Santa Cruz Technologies (Santa Cruz, CA, USA). The Tubulin (21485) antibody was from Cell Signaling Technology (Boston, MA, USA).

### Cell extracts and immunoblots

Cell extracts were prepared and immunoblots were performed as described [32]. Briefly, cell extracts were prepared in Triton Lysis Buffer [20 nM Tris–HCl, pH 7.4, 150 nM NaCl, 2 mM EDTA, 10% glycerol, 2 mM DTT, 1 mM PMSF, protease cocktail (Millipore-Sigma, Burlington, MA, USA), phosphatase inhibitor cocktail (Millipore-Sigma, Burlington, MA, USA)]. Protein transfer was performed in a buffer containing 20% methanol and the blots were probed with primary antibodies. Bands were visualized by enhanced chemiluminescence after incubation with species-matched secondary antibodies.

### siRNA knockdown

Control siRNA (211631978) and siRNAs targeting BRD2 (mm.Ri.Brd2.13.1), BRD3 (mm.Ri.Brd3.13.1), and BRD4 (mm.Ri.Brd4.13.1) were purchased from Integrated DNA Technology (Coralville, IA, USA). For some experiments, a pool of siRNAs targeting mouse Brd4 (M-041493), mouse BRD2 (M-043404), and a non-targeting siRNA (5′-UUCUCCGAACGUGUCACGU-3′) were obtained from Dharmacon (Lafayette, CO, USA). Transfection using Dharmafect 1 was performed on cells in growth media according to manufacturer’s instructions. After 48 to 72 h, growth media was replaced with differentiation media and cells were then harvested 48 h later.

### Co-immunoprecipitation studies

HEK 293T cells were transfected with FLAG-tagged MITF construct using Lipofectamine LTX (Invitrogen, Carlsbad, CA, USA). Cells were harvested 48 h post-transfection. For endogenous co-immunoprecipitations, Melb-a cells were differentiated and harvested after 6 h. Co-immunoprecipitations were performed as previously described [26]. Briefly, cell extracts were prepared in Triton lysis buffer and precleared for 30 min. 293T cells were transfected FLAG-tagged MITF were incubated with anti-FLAG M2 agarose (A2220) or normal mouse IgG agarose (AO919) (Millipore-Sigma, Burlington, MA, USA) Lysates from Melb-a cells were incubated overnight with an antibody to MITF, BRD2, BRD4, or a species-matched control antibody which was either normal mouse IgG (sc2025) or normal rabbit IgG (sc2027) (Santa Cruz Technologies). The lysates were then incubated for 4 h with Protein G or A Sepharose (GE Healthcare Life Sciences, Marlborough, MA, USA), and the beads washed three times with Triton Lysis Buffer. Immunocomplexes were eluted at 50 °C for 20 min in SDS loading buffer.

### Chromatin immunoprecipitation studies (ChIPs)

Chromatin immunoprecipitation studies were performed with a control rabbit IgG, BRD2, or BRD4 antibody as previously described [32]. Briefly, cells were cross-linked with formaldehyde (1% final concentration) for 10 min at room temperature in culture media. The reaction was then stopped by adding glycine to a final concentration of 125 mM. Cells were washed twice with phosphate buffered saline and nuclei were isolated in a buffer containing 50 mM HEPES–KOH, pH 7.5, 140 mM NaCl, 1 mM EDTA, 10% glycerol, 0.5% NP40, 0.25% TritonX, and protease inhibitors. Nuclei were washed with a buffer containing 10 mM Tris–HCl, pH 8.0, 200 mM NaCl, 1 mM EDTA, 0.5 mM EGTA and protease inhibitors. Nuclei were sonicated in 20 s pulses for 4 min in a buffer containing 10 mM Tris–HCl, pH 8.0. 100 mM NaCl, 9 mM EDTA, 0.5 mM EGTA, 0.1% Na-deoxycholate, 0.5% *N*-lauroylsarcosine and 1.1% Triton X-100. Complexes were incubated overnight with antibodies and then incubated with Protein A or G Sepharose (GE Healthcare Life Sciences, Marlborough, MA, USA). Complexes were washed five times in RIPA buffer (50 mM HEPES, 500 mM LiCl, 0.1 mM EDTA, 1.0% NP-40 and 0.7% Na-deoxycholate) and once with TE containing 50 mM NaCl. The immune complexes were eluted with 50 mM Tris [pH 8.0], 10 mM EDTA and 1.0% SDS and heated overnight at 65 °C to reverse crosslinks. DNA was then purified by digesting with proteinase K followed by phenol chloroform extraction and ethanol precipitation.

Primers used were as follows:

Murine *Tyr* promoter: Forward: 5′-AGT CAT GTG CTT TGC AGA AGA T-3′ and Reverse: 5′-CAG CCA AGA ACA TTT TCT CCT T-3′.

Murine *Tyrp1* promoter: Forward: 5′-GCA AAA TCT CTT CAG CGT CTC-3′ and Reverse: 5′-AGC CAG ATT CCT CAC ACT GG-3′.

Murine *IgH* enhancer: Forward: 5′-GCC GAT CAG AAC CAG AAC ACC-3′ and Reverse: 5′- TGG TGG GGC TGG ACA GAG TGT TTC-3′.

### Chromatin accessibility (FAIRE analysis)

The formaldehyde-assisted isolation of regulatory elements (FAIRE) assay was performed as previously described [26]. Briefly, cells were cross-linked with 1% formaldehyde for 6 min at room temperature, then nuclei were isolated and sonicated as described for ChIPs. Chromatin was extracted twice with phenol/chloroform, back extracted with TE and then extracted with chloroform. The aqueous phase was then heated at 65 °C overnight to reverse crosslinks. DNA was purified as described for ChIPs. Primers used were the same as for ChIP studies.

### Statistical analysis

Statistical significance was calculated by the Student’s *t* test using Graphpad Prism.

## Results

### Treatment of Melb-a melanoblasts with (+)JQ1 inhibits visible pigmentation and melanin synthesis

**Fig. 1:**
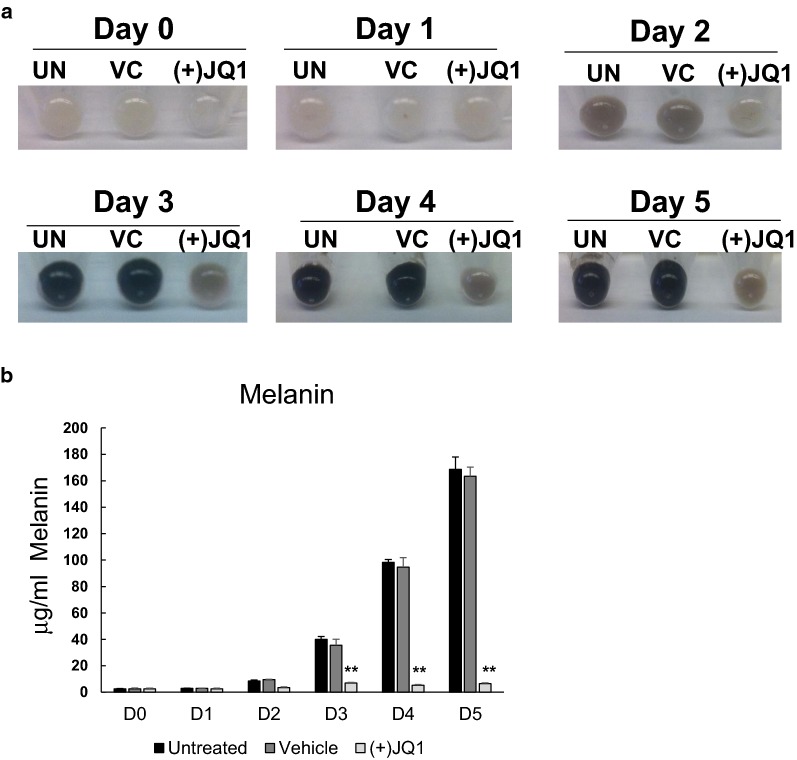
BET inhibition suppresses melanin synthesis. Melb-a cells were differentiated for the indicated number of days in the presence or absence of the BET bromodomain inhibitor (+)JQ1 (500 nM). Cells were pelleted and **a** photographed or **b** subjected to a melanin assay (*UN* untreated, *VC* vehicle treated). The results are the average three independent experiments. Standard error bars are shown. Statistically significant differences between VC and (+)JQ1 are shown (*p < 0.05, **p < 0.01, ***p < 0.001)

Melb-a cells are unpigmented mouse melanoblasts that can be induced to differentiate into pigmented melanocytes over the course of several days [33]. To determine if BET proteins regulate the process of melanogenesis, Melb-a melanoblasts were induced to differentiate in the presence or absence of the active stereoisomer of the BET protein inhibitor (+)JQ1. As previously reported, in the absence of (+)JQ1, Melb-a cells became progressively pigmented when they were induced to differentiate and synthesize melanin [26]. However, treatment with (+)JQ1 inhibited visible pigmentation (Fig. 1a) and melanin synthesis (Fig. 1b). We found that melanogenesis in Melb-a cells was similarly inhibited by the BET inhibitor, PFI-1 (Additional file 1: Fig. S1).

### The inhibitory effects of (+)JQ1 on pigmentation and melanin synthesis are dose dependent and reversible

**Fig. 2:**
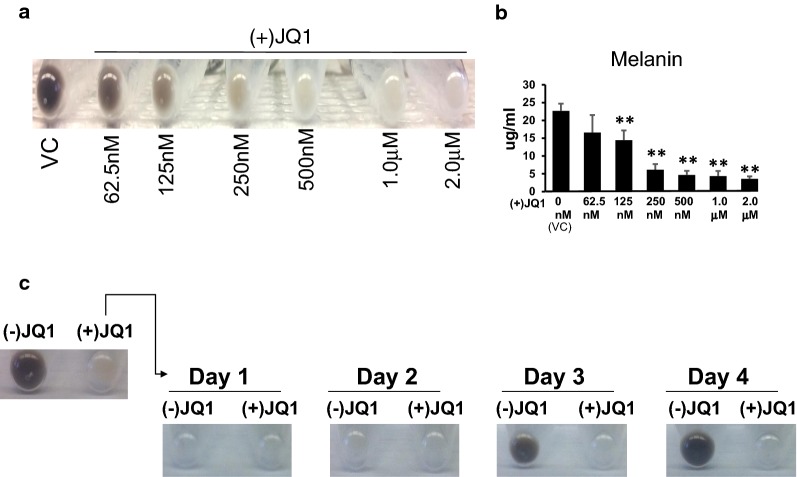
The effects of BET inhibition on melanin synthesis is dose dependent and reversible. **a** Melb-a cells were differentiated for 48 h in the presence of vehicle (VC) or (+)JQ1 at the indicated concentrations. Cells were pelleted and photographed. **b** Melanin assays were performed on cells shown in **a**. The results are the average of three independent experiments. Standard error bars are shown. Statistically significant differences between VC and different concentrations of (+)JQ1 are shown (*p < 0.05, **p < 0.01, ***p < 0.001). **c** Melb-a cells were differentiated for 72 h in the presence of inactive (−)JQ1 or active (+)JQ1 (500 nM) (top). Cells from (+)JQ1 treated samples were washed and passaged into differentiation media containing inactive (−)JQ1 or active (+)JQ1 and further cultured for the indicated number of days (bottom)

We tested the dose-dependent effects of (+)JQ1 on melanogenesis by exposing Melb-a cells to (+)JQ1 concentrations ranging from 0 to 2 μM. Increasing concentrations of (+)JQ1 had progressively inhibitory effects on visible pigmentation (Fig. 2a). When we quantified melanin synthesis by normalizing to cell counts, we found that 125 nM (+)JQ1 was the lowest dose to significantly inhibit melanin synthesis and that the maximum inhibitory effect was achieved with 500 nM (+)JQ1.

Several studies indicate that the effects of BET inhibitors on biological processes are reversible once the drug is removed [34, 35]. To determine the durability of BET inhibition on melanogenesis, we induced differentiation of Melb-a cells in the presence of an inactive enantiomer of JQ1, (−)JQ1 or the active isomer (+)JQ1. After 72 h, the cells that were induced in the presence of (−)JQ1 were visibly pigmented while those induced in the presence of (+)JQ1 were not (Fig. 2c, top). We then split (+)JQ1-treated cells into two plates, one of which was cultured in the presence of (+)JQ1 and the other in the presence of (−)JQ1. We found that pigmentation was restored 3 days after replacement of (+)JQ1 with inactive (−)JQ1, while cells that were maintained in media with (+)JQ1 remained unpigmented (Fig. 2c, bottom). Thus, the inhibitory effect of BET inhibition on melanogenesis lasted several days, but was ultimately reversible after drug removal.

We next investigated whether BET inhibitors can suppress melanogenesis in normal human epidermal melanocytes (NHEMs). Unlike Melb-a melanoblasts which are initially unpigmented and can be induced to synthesize melanin, NHEMs are pigmented and constitutively produce melanin. We found that treatment of NHEMs with (+)JQ1 resulted in time-dependent de-pigmentation (Additional file 2: Fig. S2). This suggests that BET proteins are required for maintaining pigmentation of differentiated melanocytes.

### (+)JQ1 inhibits Melb-a proliferation

**Fig. 3:**
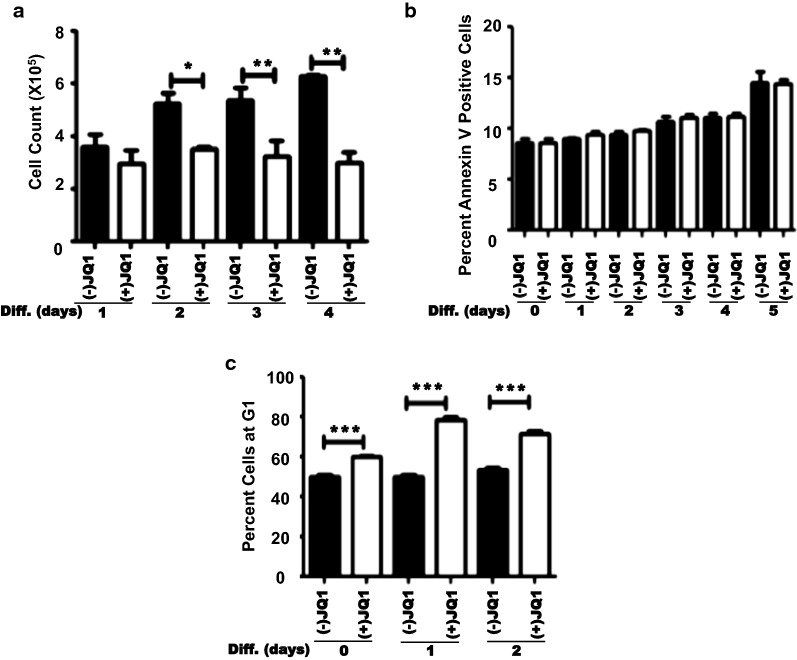
Melb-a cells treated with (+)JQ1 accumulate in the G1 phase of the cell cycle. **a** Melb-a cells were differentiated for the indicated number of days with 500 nM inactive (−)JQ1 or 500 nM active (+)JQ1 and cultured for the indicated number of days. **a** Cell counts. **b** Cells were stained with Annexin V and sorted by flow cytometry. **c** Cells were stained with propidium iodide and sorted by flow cytometry. The results are the average of three independent experiments. Standard error bars are shown. Statistically significant differences between (−)JQ1 and (+)JQ1 treated samples at each time point are shown (*p < 0.05, **p < 0.01, ***p < 0.001)

We found treatment with (+)JQ1 decreased Melb-a proliferation during differentiation (Fig. 3a). To determine if the effects on proliferation were due to cell death or changes in the cell cycle, we differentiated Melb-a cells in the presence of (−)JQ1 or (+)JQ1 and evaluated both apoptosis and cell cycle progression at several time-points. We found no significant difference in apoptosis between (−)JQ1 and (+)JQ1 treated cells at any time point during the differentiation process (Fig. 3b). However, there was a significant increase in the number of cells in the G1 phase when they were induced to differentiate in the presence of (+)JQ1 (Fig. 3c). These results suggest that BET inhibition during melanocyte differentiation suppresses proliferation primarily by promoting G1 arrest.

### BET inhibition alters the expression of melanocyte and melanoma-specific gene expression

To determine the effects of (+)JQ1 on gene expression, we performed RNA-seq of Melb-a cells that were differentiated in the presence or absence of (+)JQ1. After 48 h, we detected widespread effects on gene expression, with 4069 significantly up-regulated genes and 4430 significantly down-regulated genes (Additional file 3: Fig. S3A). GO-seq analysis indicated that nucleoside and protein binding, RNA processing and modification, and metabolic processes were enriched (Additional file 3: Fig. S3B, C). Kegg analysis indicated that there was significant down-regulation of metabolic pathways and up-regulation of ribosome and spliceosome-related gene expression (Additional file 3: Fig. S3D, E). Interestingly, Kegg analysis indicated up-regulation of cell cycle components (Additional file 3: Fig. S3) including *Rb1*, *Ink4b* (p15) and *Cdkn1b* (p27). We also noted a decrease in cyclin D1 expression. A change in the expression of these cell cycle regulators might explain how (+)JQ1 promotes G1 arrest (Fig. 3c).

**Fig. 4:**
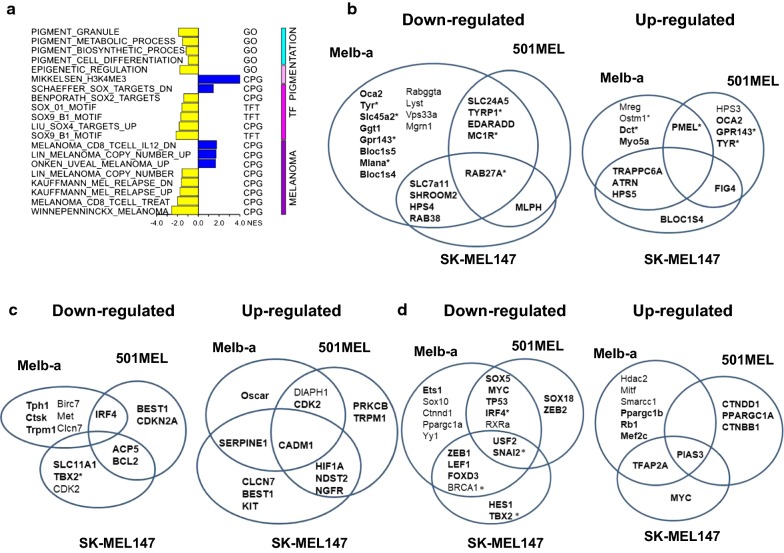
BET inhibition alters melanocyte differentiation and melanoma-specific gene expression. Melb-a cells were differentiated in the presence of vehicle or 500 nM (+)JQ1. RNA from three biological replicates was subjected to RNA-seq. Differential gene expression between vehicle (DMSO) and 500 nM (+)JQ1 was determined from RNA-seq data (*p* < 0.05, *q* < 0.25). **a** Upon small molecule inhibition, pathways relevant to pigmentation and melanoma proliferation and resistance were identified and quantified using normalized enrichment scores (NES) with *p* < 0.05 and *q* < 0.25. **b**–**d** Venn diagrams showing overlap of gene expression relevant to melanocyte differentiation between BET inhibited Melb-a cells and BET inhibited melanoma cells. Bold text indicates differential gene expression > 1.5. **b** Down-regulated (left) and up-regulated (right) pigment genes. Asterisks indicate that they are MITF target genes. **c** Down-regulated (left) and up-regulated (right) transcriptional regulators of melanocyte differentiation. Asterisks indicate that they are MITF target genes. **d** Additional MITF target genes involved in proliferation, mast cell and osteoclast differentiation that are down-regulated (left) or up-regulated (right)

Additional pathway analysis focused on melanocyte and melanoma relevant pathways revealed major cellular networks of melanogenesis, cellular differentiation, epigenetic regulation, and transcriptional regulation to be significantly enriched (Fig. 4a). Specifically, transcriptional networks controlled by SOX family members were down-regulated as were pathways associated with melanoma resistance and disease progression.

To understand the molecular basis for the observed effects of (+)JQ1 on pigmentation and to delineate a role for BET proteins in the regulation of melanogenesis, we interrogated the effects of (+)JQ1 on the expression of 49 curated “color” genes [36]. This list includes curated “color” genes that either directly regulate melanin synthesis or proteins involved in melanosome construction and transport (excluding transcriptional and systemic regulators). We analyzed the transcriptional effects of BET inhibition on “color” genes in Melb-a cells and compared this data to a previously reported transcriptome analysis of (+)JQ1-treated 501Mel and SK-Mel147 melanoma cells [37]. BET inhibition suppressed the expression of nearly half the “color” genes in Melb-a cells, and a smaller number (6 genes) in 501MEL and (6 genes) in SK-MEL147 melanoma cells (Fig. 4b, left). However, some “color” genes were up-regulated in BET inhibited Melb-a cells, a number similar to that in 501MEL and in SK-MEL147 melanoma cells (Fig. 4b, right). Interestingly, there was greater overlap in gene regulation by BET inhibition between Melb-a and either 501MEL or SK-MEL147 melanoma cells than between 501MEL and SK-MEL147. RAB27A was the only down-regulated gene amongst all three cell lines. Thus, BET inhibition has both overlapping and distinct effects on the expression of intrinsic regulators of pigmentation, which are markers of terminal melanocyte differentiation. These results suggest that (+)JQ1 potently inhibits differentiation of melanoblasts and mildly affects the differentiation status of melanoma cells.

Interrogation of a larger list of pigment-related genes (379 eGenes) [38] (including both positive and negative regulators of pigmentation) showed a similar trend with the expression of almost 50% of eGenes being differentially regulated as a result of BET inhibition in Melb-a cells and 20–30% of eGenes in melanoma cells (Additional file 4: Table S1). We noticed differential regulation of several transcriptional regulators and chromatin remodeling enzymes that impact pigmentation in all three cell lines (Fig. 4c). IRF4, a transcriptional regulator associated with freckles, blue eyes, and brown hair, was down-regulated in both Melb-a and 501MEL cells [39]. SNAI2, a transcriptional repressor involved in melanocyte migration and associated with pigmentary disturbances in some types of Waardenburg syndrome, was down-regulated in all three cell lines [40]. Interestingly, expression of the master regulator of melanocyte differentiation, *Mitf*, was modestly (< 1.5 fold) but significantly up-regulated in Melb-a cells. However, expression of *PIAS3*, a repressor of MITF activity [41], was strongly up-regulated (> 1.5 fold) in all three cell lines, suggesting that BET inhibition might suppress MITF activity. This could explain why many MITF target genes were down-regulated while *Mitf* expression was only slightly up-regulated.

Many of the pigment genes affected by BET inhibition are MITF target genes (Fig. 4b, denoted by asterisks). MITF also regulates genes involved in melanocyte proliferation and survival [42] as well as mast cell [43] and osteoclast function [44]. We found that BET inhibition suppressed expression of 36% of validated MITF target genes [45] in Melb-a cells, half as many as in melanoma cells. BET inhibition increased expression of approximately 22% of the MITF target genes in all three cell lines, with overlapping effects on expression of genes involved in proliferation, survival, mast cell, and osteoclast function (Fig. 4d). There was also a high degree of overlap between genes affected by BET inhibition (Additional file 5: Table S2) and genes that were differentially regulated by MITF in melanoma cells (50% in Melb-a cells and 40% in 501Mel and SK-MEL147 cells) [42].

### BET inhibition decreases TYR and TYRP1 expression in Melb-a and normal human epidermal melanocytes

**Fig. 5:**
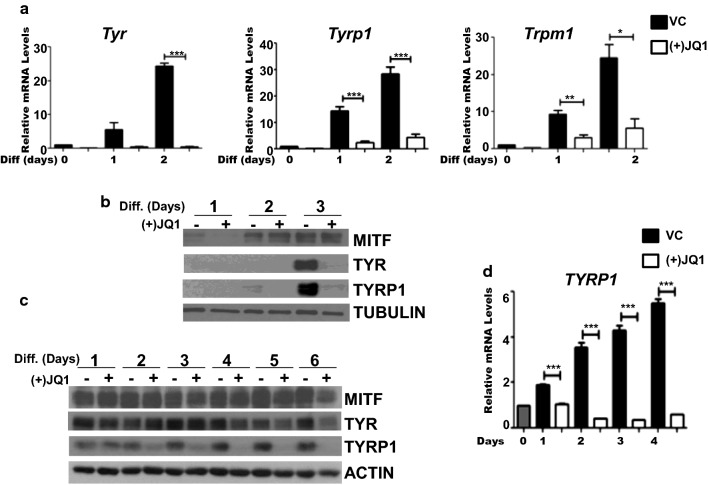
Melanocyte differentiation gene expression is suppressed by BET inhibition. **a** Melb-a cells were differentiated in the presence of vehicle (VC) or 500 nM (+)JQ1. Cells were harvested at the indicated time-points and subjected to qRT-PCR. *Tyr*, *Tyrp1*, or *Trpm1* levels were normalized to that of *Rpl7*. The data are the average of three independent experiments. Standard error bars are shown. Statistically significant difference between VC and (+)JQ1 treated samples at each time point are shown (*p < 0.05, **p < 0.01, ***p < 0.001). **b** Protein extracts from cells cultured in the absence (vehicle treated) or presence of (+)JQ1 were subjected to immunoblot analysis using the indicated antibodies. The figure is representative of three or more experiments. **c** Neonatal human epidermal melanocytes (NHEMs) were cultured in the absence (vehicle treated) or presence of 500 nM (+)JQ1 for the indicated number of days. Harvested cells were subjected to immunoblot analysis using the indicated antibodies. The figure is representative of three or more experiments. **d** NHEMs were cultured as in **c** and then subjected to qRT-PCR. The *TYRP1* level was normalized to that of *RPL9*. The data are the average of three independent experiments. Standard error bars are shown. Statistically significant difference between VC and (+)JQ1 treated samples at each time point are shown (*p < 0.05, **p < 0.01, ***p < 0.001)

To further investigate the role of BET proteins in the regulation of MITF target genes, we assayed the effects of (+)JQ1 treatment on gene expression during a time course of melanocyte differentiation. We confirmed earlier reports of a time-dependent increase in *Tyr*, *Tyrp1*, and *Trpm1* mRNA levels during melanoblast differentiation (Fig. 5a) [26]. However, treatment of melanoblasts with (+)JQ1 inhibited the expression of these genes at all time-points (Fig. 5a) and prevented accumulation of TYR and TYRP1 enzymes, which are required for melanin synthesis (Fig. 5b). In contrast, MITF protein levels were not noticeably affected by treatment with (+)JQ1 (Fig. 5c).

Similarly, the depigmenting effects of (+)JQ1 on human melanocytes (Additional file 2: Fig. S2) corresponded with decreased protein levels of TYRP1, occurring day 1 after treatment and decreased expression of TYR and MITF occurring over a period of 6 days (Fig. 5c). Treatment with (+)JQ1 suppressed *TYRP1* at the mRNA level (Fig. 5d), but did not have a significant effect on *TYR* nor *MITF* mRNA levels during the time period tested (data not shown). Thus, BET inhibition of NHEMs suppressed expression of genes required for melanin synthesis, but the kinetics and gene-specific effects were different from that observed in differentiating Melb-a melanoblasts.

### BRD4 and BRD2 are required for expression of melanocyte-specific genes

**Fig. 6:**
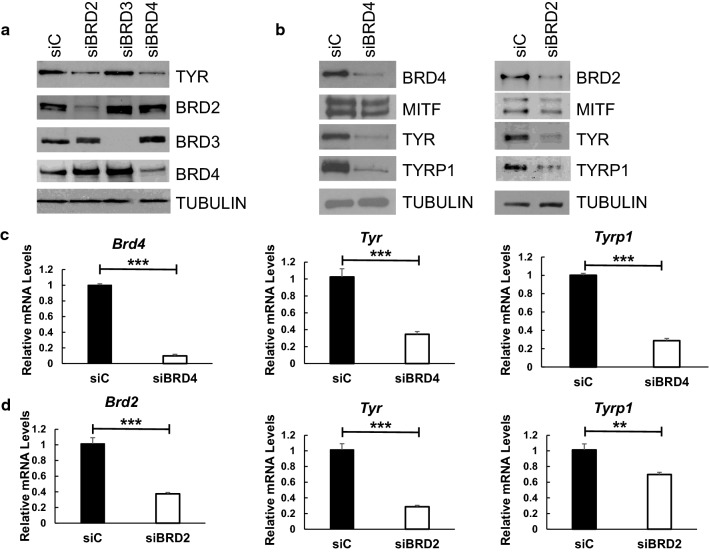
Depletion of BRD4 or BRD2 in Melb-a cells suppresses expression of melanocyte-specific genes. **a** Melb-a cells were transfected with control siRNA or siRNA that targets BRD2, BRD3, or BRD4, then differentiated for 48 h. Immunoblot analysis was performed with the indicated antibodies. The immunoblot is representative of two or more experiments. **b** Melb-a cells were transfected with a control (siC) or a pool of siRNAs that targets BRD4 or BRD2 (distinct from those used in **a**) then differentiated for 48 h. Immunoblot analysis was performed with the indicated antibodies. The immunoblot is representative of two or more experiments. **c**, **d** BRD4 or BRD2 depleted cells treated as described in **b** were subjected to qRT-PCR. *Brd4*, *Brd2*, *Tyr*, and *Tyrp1* levels were normalized to that of *Rpl7*. The data in **c** and **d** are the average of three independent experiments. Standard error bars are shown. Statistically significant differences between siC, siBRD4, and siBRD2 transfected cells are shown (*p < 0.05, **p < 0.01, ***p < 0.001)

To determine if any or all of the BET protein family members are required for expression of melanocyte-specific genes, we transfected Melb-a cells with control siRNA or siRNAs that target BRD2, BRD3, or BRD4. We found that down-regulation of BRD2 or BRD4, but not BRD3 decreased TYR protein levels (Fig. 6a). Since BRD4 has been implicated in several cellular differentiation processes [34, 46–49], we investigated its role in melanocyte differentiation more thoroughly. In order to control for potential off-target effects, we used a second siRNA to deplete BRD4 from Melb-a cells. There was a decrease in TYR and TYRP1 at the protein level (Fig. 6b) and at the mRNA level (Fig. 6c). Because down-regulation of BRD2 also affected TYR protein levels, we used a second siRNA to deplete BRD2 and investigate its role in melanocyte investigation. Like BRD4, we found that BRD2 depletion inhibited TYR and TYRP1 expression at both the protein (Fig. 6b) and at the mRNA levels (Fig. 6d).

### BRD4 and BRD2 occupy MITF-binding sites and are associated with open chromatin structure at *TYR* and *TYRP1* loci

**Fig. 7:**
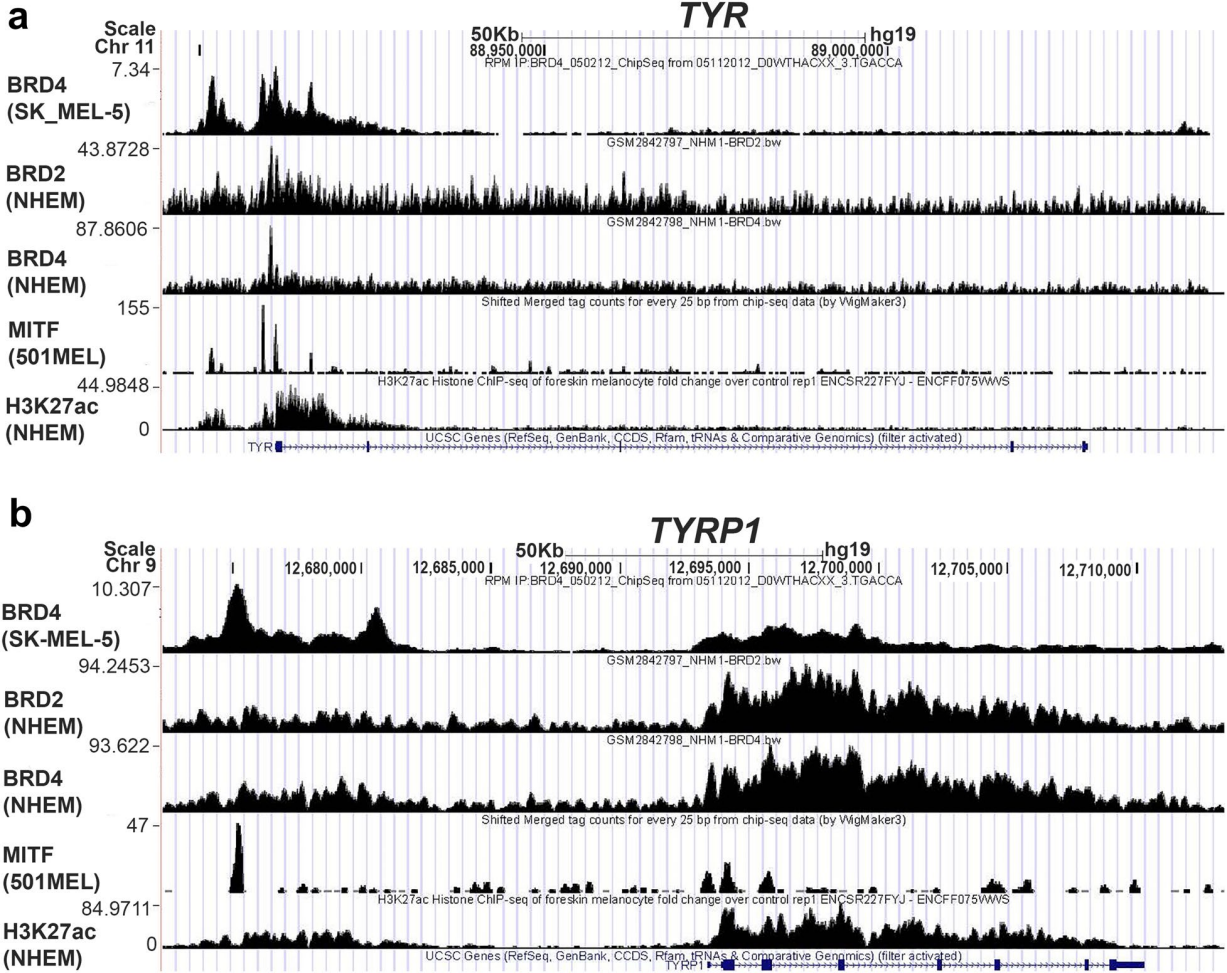
BRD4 and BRD2 binding overlap H3K27ac and canonical MITF-binding sites at melanocyte-specific loci. Publicly available ChIP-seq data in normal human melanocytes, SK-MEL-5 melanoma cells (GSM1968282), 501 melanoma cells (GSM1517751), and neonatal human epidermal melanocytes (NHEM) (GSM2842798) with the indicated antibodies at the **a***TYR* and **b***TYRP1* loci

Since *TYR* and *TYRP1* are MITF target genes, we interrogated previously published ChIP-seq datasets to determine if BRD4 and MITF co-occupy MITF-binding sites at the *TYR* and *TYRP1* loci in melanocytes and melanoma cells. BRD4 and BRD2 peaks overlapped MITF and histone H3 lysine 27 acetylation (H3K27ac) peaks, at the *TYR* promoter region and at an upstream enhancer region and promoter of *TYRP1* (Fig. 7a, b).

We also interrogated other representative MITF target genes that were either down-regulated (Additional file 6: Fig. S4) or up-regulated (Additional file 7: Fig. S5) by (+)JQ1. While BRD4 and BRD2 peaks as well as H3K27ac peaks overlapped MITF-binding sites at the majority of these representative genes, in both normal human melanocytes and melanoma cells, there were some MITF sites such as within the *DCT*, *PIAS3*, *SLC45A2*, and *IRF4* loci where BRD4 binding was observed in SK-MEL-5 melanoma cells but not normal human melanocytes (Additional file 7: Fig. S5). Differences in BET protein binding in different cell lines could explain some of the cell line-specific effects of (+)JQ1 on gene expression. Interestingly, *PIAS3*, which was highly up-regulated by (+)JQ1, had several BRD2 peaks which did not overlap the MITF-binding site nor H3K27ac-rich regions which could potentially have an inhibitory effect on transcription (Additional file 7: Fig. S5).

While it was possible to obtain overlapping BRD4, BRD2, and MITF peaks on representative genes in different melanocytes and melanoma cells, we could not find BRD4, BRD2, and MITF ChIP-seq data for the same melanocyte or melanoma cell line to reliably quantify the overlap. However, when we compared Encode data for the K257 leukemia cell line using the GSM2527370 data for MITF and the GSM2700494 data for BRD4, we found 6937 MITF peaks and 8509 BRD4 peaks, with 1204 overlapping between MITF and BRD4. Using the peakPermTest function, we determined a *p*-value of 0.099 indicating highly significant overlap. This suggests that MITF and BRD4 are also likely to interact in melanocytes and melanoma cells. However, additional ChIP-seq studies will be needed to determine the extent of the MITF/BRD4/BRD2 overlap on melanocyte-specific loci.

**Fig. 8:**
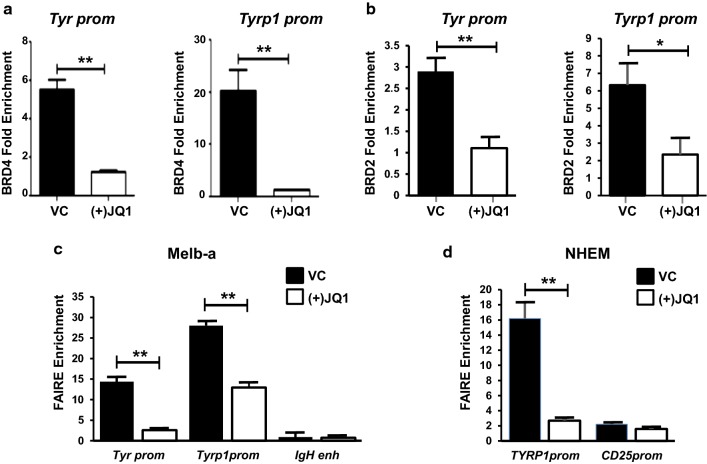
BRD4 and BRD2 occupancy at MITF-binding sites is associated with chromatin accessibility in Meb-a cells. **a**, **b** Melb-a cells were differentiated for 24 h in the presence of vehicle (VC) or 500 nM (+)JQ1. Cells were cross-linked and control IgG or an antibody to BRD4 or BRD2 was used for ChIP. BRD4 and BRD2 binding to the MITF-binding sites to the promoter regions of *Tyr* and *Tyrp1* was assayed by qPCR. BRD4 and BRD2 enrichment is shown relative to IgG enrichment and normalized to a non-specific region (*IgH* enhancer). The data are the average of three independent experiments. Standard error bars are shown statistically significant differences between VC and (+)JQ1 treated samples are shown (*< 0.05, **p < 0.01, ***p < 0.001). **c** Melb-a cells were differentiated as described in **a**, **b**. FAIRE enrichment at the MITF-binding sites in the *Tyr* and *Tyrp1* promoters was determined by qPCR. Enrichment at the *IgH* enhancer is shown as a negative control. The data are the average of two independent experiments performed in triplicate Statistically significant differences between VC and (+)JQ1-treated samples are shown (*< 0.05, **p < 0.01, ***p < 0.001). D. NHEMs were treated with 500 nM inactive (−)JQ1 or 500 nM active (+)JQ1 for 48 h, then harvested for FAIRE. FAIRE enrichment at the MITF-binding site in the *TYRP1* promoter was determined by qPCR. FAIRE enrichment at the *CD25* promoter is shown as a negative control. The data is the average of two independent experiments performed in triplicate Statistically significant differences between (−)JQ1 and (+)JQ1-treated samples are shown (*< 0.05, **p < 0.01, ***p < 0.001)

Consistent with the ChIP-seq data showing overlapping peaks in human melanocytes and melanoma cells, we found that both BRD4 and BRD2 were bound to the *Tyr* and *Tyrp1* promoters in Melb-a cells and that (+)JQ1 disrupted binding (Fig. 8a, b). Furthermore, FAIRE analysis indicated that (+)JQ1 also decreased chromatin accessibility at the MITF-binding sites in the *Tyr* and *Tyrp1* promoters in Melb-a cells and in the *TYRP1* promoter in human melanocytes (Fig. 8c, d).

**Fig. 9:**
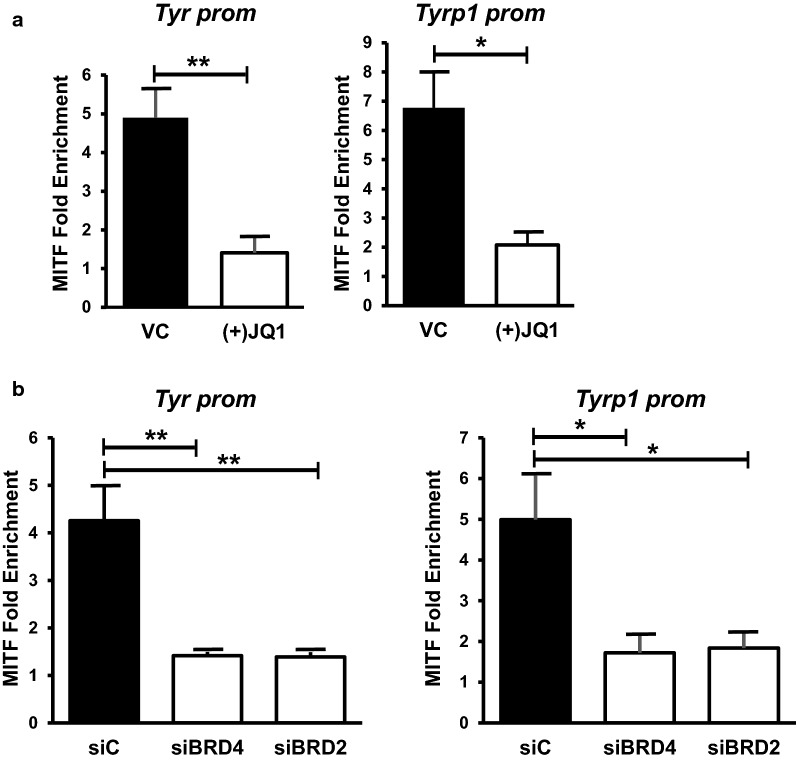
BRD4 and BRD2 promote MITF occupancy at the *Tyr* and *Tyrp1* promoters. **a** Melb-a cells were differentiated and cross-linked as described in Fig. 8. ChIPs were performed with a control IgG or an antibody to MITF. MITF enrichment is shown relative to IgG enrichment and normalized to a non-specific region (*IgH* enhancer). The data are the average of three independent experiments. Standard error bars are shown. Statistically significant differences between (−)JQ1 and (+)JQ1 treated samples are shown (*< 0.05, **p < 0.01, ***p < 0.001). **b** Melb-a cells were transfected with control siRNA (siC) or a pool of siRNAs that target either BRD4 or BRD2. Cells were differentiated for 24 h then harvested for ChIPs. MITF enrichment is shown relative to IgG enrichment and normalized to a non-specific region (*IgH* enhancer). The data are the average of two independent experiments performed in triplicate. Standard error bars are shown. Statistically significant differences between siC-transfected cells and siBRD4 and siBRD2-transfected cells are shown (*< 0.05, **p < 0.01, ***p < 0.001)

We next determined that the decrease in chromatin accessibility at the *Tyr* and *Tyrp1* promoters in (+)JQ1 treated cells was associated with a reduction in MITF binding on the *Tyr* and *Tyrp1* promoters in (+)JQ1 treated cells (Fig. 9a). Furthermore, depletion of either BRD4 or BRD2 also decreased MITF binding on these promoters (Fig. 9b), suggesting that these BET proteins facilitate MITF binding to the promoters of these melanocyte-specific genes.

### BRD4 and BRD2 interact with MITF

**Fig. 10:**
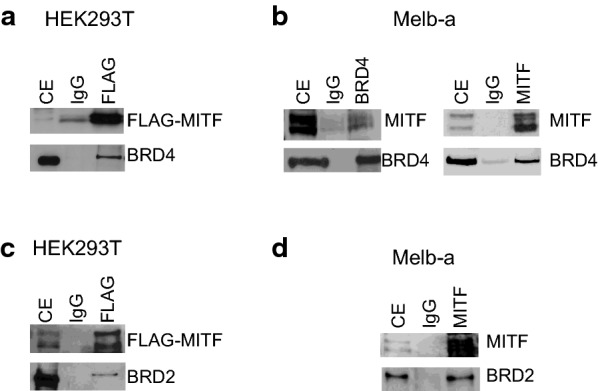
MITF co-immunoprecipitates with BRD4 and BRD2. **a**, **c** A plasmid containing FLAG-tagged MITF was transfected into HEK293T cells. Cell extracts were immunoprecipitated with control IgG or FLAG antibody. Immunoblot analysis was performed with the indicated antibodies. (*CE* cell extract). The figures are representative of two or more experiments. **b**, **d** Cell extracts from Melb-a cells were immunoprecipitated with control IgG or antibodies to BRD4, BRD2, or MITF. Immunoblot analysis was performed with the indicated antibodies. (*CE* cell extract). The figures are representative of two or more experiments

To assay for physical interactions between BRD4, BRD2, and MITF, HEK293T cells were transfected with epitope-tagged (FLAG) MITF and co-immunoprecipitation was performed with either an antibody to BRD4 or to BRD2. Endogenous BRD4 co-immunoprecipitated with transfected MITF in HEK293T cells (Fig. 10a). We also detected interactions between endogenous BRD4 and MITF in Melb-a cells (Fig. 10b). BRD2 also co-immunoprecipitated with MITF in HEK293T cells (Fig. 10c) and in Melb-a cells (Fig. 10d).

## Discussion

Small molecule inhibitors of the bromodomain and extra-terminal (BET) class of epigenetic regulators have been extensively studied for their anticancer activities, quickly advancing to human clinical trials. These inhibitors selectively bind to bromodomains of the BET family of proteins and disrupt binding to chromatin, thereby altering gene expression profiles that impact cancer cells. However, these inhibitors also affect normal biological processes. Therefore, it is critical to delineate the role of each BET protein in non-cancerous cells in order to more effectively utilize these drugs and to minimize toxicities [50].

Bromodomain and extra-terminal proteins regulate transcriptional networks required for normal organismal development, stem cell maintenance, cellular differentiation, and immune function in vivo. Mice with homozygous disruption of either *Brd2* or *Brd4* die during embryogenesis [51, 52]. BRD4 is required for maintaining the self-renewal and pluripotency of stem cells [53, 54] and BRD2 for lineage specification [55]. Inducible knockdown of BRD4 by RNAi interferes with normal hematopoiesis and leads to epidermal hyperplasia and stem cell depletion [35]. BRD4 disruption in myeloid cells compromises innate immunity [56]. Furthermore, BRD4 is required for muscle and brown adipose tissue development [47]. BET proteins also promote erythroid, osteoblast, muscle, and adipocyte differentiation [34, 46, 48, 49]. However, the function of BET proteins in melanocyte differentiation had not been investigated prior to this study.

Our findings reveal that inhibiting BET proteins with (+)JQ1 in melanoblasts suppresses differentiation, resulting in a striking decrease in visible pigmentation and melanin synthesis. Importantly, (+)JQ1 treatment resulted in reduced expression of *Tyr* mRNA and protein in differentiating Melb-a melanoblasts*. Tyr* is the gene encoding the rate-limiting enzyme in melanin synthesis, the loss of which results in a complete failure to synthesize melanin and is manifested as oculocutaneous albinism in individuals with *TYR* mutations [57]. In addition to the quantity of melanin, pigmentation is determined by the type of melanin, size, number, and structure of melanosomes [21]. We found that BET inhibition suppressed the expression of additional pigmentation genes regulating these melanocyte functions. The mRNA levels of many transcriptional regulators that affect the level of pigmentation, including *Sox10* [58], *Ets1* [59], and *Irf4* [39], were also reduced while the expression of *Tfap2* was strongly increased in both Melb-a melanoblasts and SK-MEL-147 melanoma cells. TFAP2, which promotes melanocyte differentiation by activating a subset of MITF target genes, is frequently lost in melanoma due to silencing by CpG methylation [60, 61]. Interestingly, the expression of *Pias3*, a potent inhibitor of MITF activity [41] was strongly up-regulated and the expression of an extensive number of MITF target genes was decreased. These findings suggest that BET inhibition prevented melanocyte differentiation downstream of *Mitf* expression. This mechanism could involve the reduction in the expression of other positive transcriptional regulators of MITF target genes or inhibition of MITF activity through the increase in the expression of PIAS3. Additionally, we considered the possibility that BET proteins are MITF coactivators and that (+)JQ1 inhibits this function.

Since pan-BET inhibitors such as (+)JQ1 disrupt the activities of all BET family members, regardless of the intended target, it is critical to delineate the role of each BET protein [50]. Therefore, we further explored the regulation of melanogenic genes by depleting single BET proteins in our Melb-a model. Our studies determined that both BRD4 and BRD2 are required for expression of two critical melanogenic genes, *Tyr* and *Tyrp1*, indicating a role for both BRD2 and BRD4 in melanocyte differentiation. Similarly, BRD2 and BRD4 are required for erythroid gene activation [48], but play opposing roles during adipogenesis [46, 47, 62].

Although the gene expression changes that resulted from treatment with (+)JQ1 obtained in our RNA-seq data suggests an extensive role for BET proteins in melanocyte differentiation, a limitation of this study is that we cannot determine which specific BET proteins mediate the transcriptional changes. Moreover, the three somatic BET proteins can have overlapping or opposing functions in the regulation of gene expression. Interestingly, transcriptional profiling of the epithelial mesenchymal transition showed that gene expression changes elicited by (+)JQ1 treatment most closely paralleled changes elicited by BRD2 depletion rather than by depletion of BRD3 or BRD4 which elicited opposite transcriptional changes as (+)JQ1 [3]. The opposing actions of different BET proteins could explain why some genes are activated while others repressed in our experiments. Future studies will employ an unbiased approach to determine which of the BET proteins play a prominent role in the activation or repression of the melanocyte differentiation program. However, since depletion of either BRD2 or BRD4 compromised tyrosinase expression, which is absolutely required for melanin synthesis, our data suggest that these two proteins play an important role in pigmentation. Therefore, we proceeded to investigate the mechanism by which they regulate melanocyte-specific gene expression.

We found that both BRD4 and BRD2 physically interact with MITF and that occupancy of both proteins overlap MITF-binding sites on MITF target promoters. Furthermore, depletion of either BRD4 or BRD2 compromised MITF recruitment to melanocyte-specific promoters. Similarly, BRD2, BRD3, and BRD4 interact with master regulators of adipocyte and erythroid differentiation [47, 63]. BET proteins interact with acetylated GATA 1 to promote erythroid differentiation and depletion of BET proteins can also compromise GATA1 recruitment to erythroid genes. Acetylation of the RelA subunit of the NF-κβ has also been demonstrated to promote interactions with BET proteins [64]. Although there are no published reports of MITF being acetylated, there are several lysine residues upstream of the basic helix loop helix domain that are predicted to be acetylation sites [65]. These are in regions of MITF that modulate its localization to the nucleus [65, 66]. Moreover, MITF like MYOD in myocytes, interacts with the histone acetyl transferase, CBP, which can affect DNA binding and activity [67, 68]. It is tempting to speculate that acetylation of these helix loop helix lineage-specific transcription factors also facilitates binding to bromodomain proteins such as BRD4. However, neither BRD4 nor BRD2 were identified in an unbiased screen of MITF interacting proteins [69]. This may indicate that BRD4/BRD2/MITF complexes are less abundant than those of other MITF-binding partners that were identified in the interactome or that the BRD4/BRD2/MITF interaction is mediated by other proteins, weaker, or more difficult to detect. Interestingly, the BRG1 component of the SWI/SNF complex which is present in the MITF interactome is also known to physically interact with BRD4 and BRD2 [8, 54]. Therefore, BRG1 or a BRG1-associated factor (BAF) could potentially mediate BRD4 and BRD2 interactions with MITF. However, additional studies will be required to determine the mechanisms by which BRD4 and BRD2 interact with MITF.

There was a less striking effect of BET inhibition on the expression of differentiation markers in melanoma cells when we compared a published data set [37] with our results in melanoblasts. While this could be due to the variations in experimental conditions, differences in differentiation status may also be a cause. In our experiments, Melb-a cells were induced to differentiate and express previously silent genes, while in melanoma cells, these genes were constitutively on or off. Our experiments on normal human melanocytes showed that BET inhibition can cause de-pigmentation by reducing expression of TYR and TYRP1 in a time-dependent fashion. Interestingly, we observed that inhibition of TYRP1 expression occurred at the mRNA and protein levels, but that inhibition of TYR occurred only at the protein level, suggesting that BET inhibition may affect TYR at a post-transcriptional level. Mechanistically, the inhibition of TYR could result from decreased TYRP1, which is known to regulate TYR protein stability [70]. The lack of a transcriptional effect on *TYR* expression by BET inhibition of differentiated melanocytes suggests that there are molecular distinctions regarding the induction of “differentiation” in melanoblasts compared with “de-differentiation” of melanocytes. This could imply that for some genes, BET proteins are required for induction but not for maintenance of gene expression. Alternatively, complete “de-differentiation” of melanocytes by BET inhibition may take longer than the duration of this study. Likewise, the observation that BET inhibition of melanoma cells had either no effect on *TYR* expression in SK-MEL-147 or increased its expression in 501MEL cells could be due to the distinct differentiation status of each cell line. In melanoma cells, the altered signaling pathways in SK-MEL-147 cells, which have an NRAS mutation and 501Mel cells, which have a BRAF mutation, could also be a reason for the differential response to BET inhibitors. Pharmacologic inhibition of mutant BRAF in melanoma cells activates melanocyte-specific gene expression [25]. Therefore, it is possible that signaling pathways can impact upon the interaction between MITF and BET proteins, possibly through regulation of MITF posttranslational modifications, such as acetylation.

## Conclusions

This study demonstrates that BET proteins play a role in melanocyte differentiation. We found that chemical inhibition of BET proteins by (+)JQ1 decreases melanin synthesis and de-regulates expression of melanocyte-specific gene expression in both normal melanocytes and in melanoma. We have elucidated a mechanism by which the BET protein, BRD4, interacts with MITF to regulate expression of genes important for melanin synthesis. Both MITF and the melanocyte differentiation program have been shown to confer aggressiveness and promote resistance to therapeutics [24, 25]. The observation that BET inhibition can alter this process can impinge upon therapeutic approaches.

## Supplementary information

**Additional file 1: Fig. S1.** Melb-a cells were differentiated for the indicated number of days in vehicle (VC) or 500 nM PFI-1. Cells were pelleted and photographed.

**Additional file 2: Fig. S2.** Neonatal human epidermal melanocytes (NHEMs) were cultured in the presence of vehicle (VC) or 500nM (+)JQ1 for the indicated number of days. Cells were pelleted and photographed.

**Additional file 3: Fig. S3.** Melb-a cells were differentiated for 48 h in the presence of vehicle or 500nM +JQ1. RNA was isolated and subjected to RNA-seq. Differential gene expression ((+)JQ1 vs. Vehicle) was determined based on the average of three biological replicates (adj. *p* < 0.05). A. Volcano plot showing down-regulated gene expression (green) and up-regulated gene expression (red). B. Gene ontology analysis of up-regulated biological processes, cellular components, molecular function. C. Gene ontology analysis of down-regulated biological processes, cellular components, molecular function. D. Kegg Pathway analysis of up-regulated pathways. E. Kegg Pathway analysis of down-regulated pathways.

**Additional file 4: Table S1.** The effect of BET inhibition on E-gene expression. E-genes that are significantly (*p *< 0.05) down-regulated (yellow) or up-regulated (blue) by BET inhibition of Melb-a, 501MEL, or SK-MEL147 cells. E-genes that are affected by BET inhibition in all three cell lines are shown in bold type.

**Additional file 5: Table S2.** The effect of BET inhibition on MITF-target gene expression. MITF target genes that are significantly (*p *< 0.05) down-regulated (yellow) or up-regulated (blue) by BET inhibition of Melb-a, 501MEL, or SK-MEL147 cells. MITF target genes that are affected by BET inhibition in all three cell lines are shown in bold type.

**Additional file 6: Fig. S4.** Publicly available ChIP-seq data in normal human melanocytes, SK-MEL-5 melanoma cells (GSM1968282), 501 melanoma cells (GSM1517751), and neonatal human epidermal melanocytes (NHEM) (GSM2842798) with the indicated antibodies at the MITF target genes that are down-regulated by (+)JQ1 treatment.

**Additional file 7: Fig. S5.** Publicly available ChIP-seq data in normal human melanocytes, SK-MEL-5 melanoma cells (GSM1968282), 501 melanoma cells (GSM1517751), and neonatal human epidermal melanocytes (NHEM) (GSM2842798) with the indicated antibodies at the MITF target genes that are up-regulated by (+)JQ1 treatment.

## Data Availability

The RNA-seq dataset has been deposited into the NCBI Sequence Read Archive (SRA) database (https://www.ncbi.nlm.nih.gov/sra/), under the Accession number PRJNA560240.
