# Supplementary figures and images for: Bromodomain and extra-terminal domain (BET) proteins regulate melanocyte differentiation
Source: Epigenetics Chromatin. 2020 Mar 10;13:14. doi: 10.1186/s13072-020-00333-z (PMC7063807; doi:10.1186/s13072-020-00333-z)

**Fig. S1**

**Day 0**

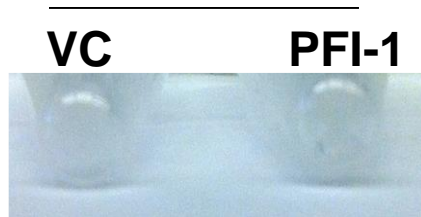

**Day 1**

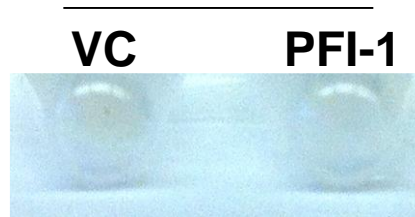

**Day 2**

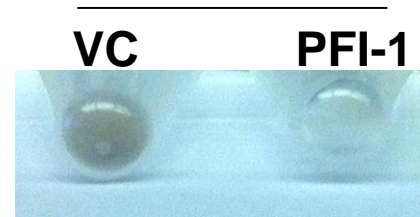

**Day 3**

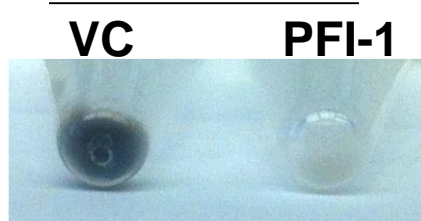

**Day 4**

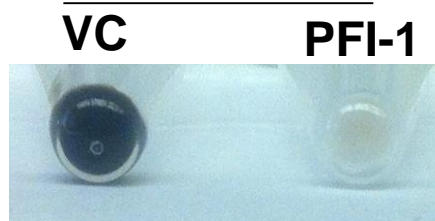

**Day 5**

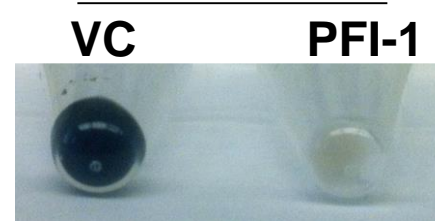

Supplement: Supplementary file 1 — Additional file 1: Fig. S1. Melb-a cells were differentiated for the indicated number of days in vehicle (VC) or 500 nM PFI-1. Cells were pelleted and photographed. [file 13072_2020_333_MOESM1_ESM.pdf]

Fig. S2

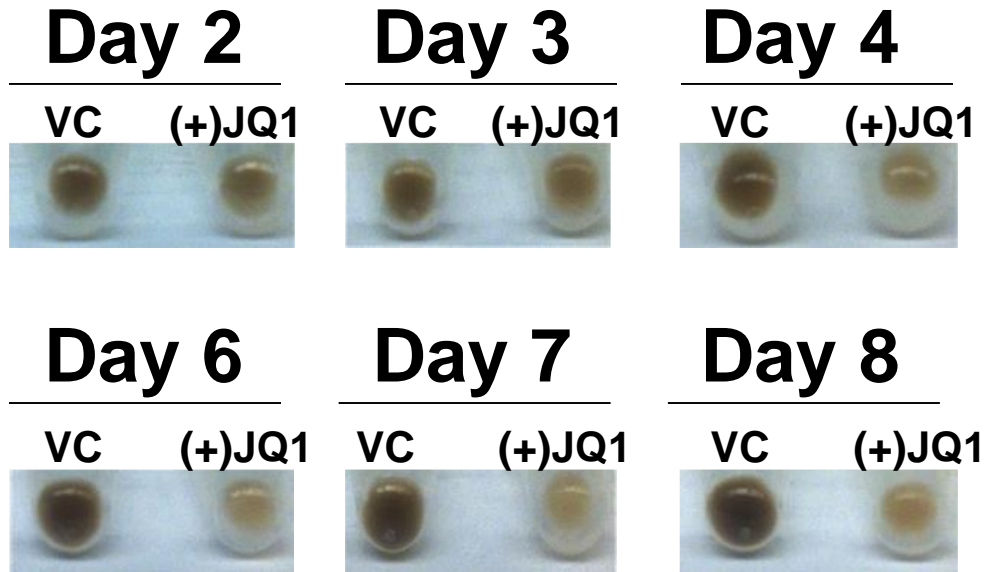

Supplement: Supplementary file 2 — Additional file 2: Fig. S2. Neonatal human epidermal melanocytes (NHEMs) were cultured in the presence of vehicle (VC) or 500nM (+)JQ1 for the indicated number of days. Cells were pelleted and photographed. [file 13072_2020_333_MOESM2_ESM.pdf]

Fig. S4

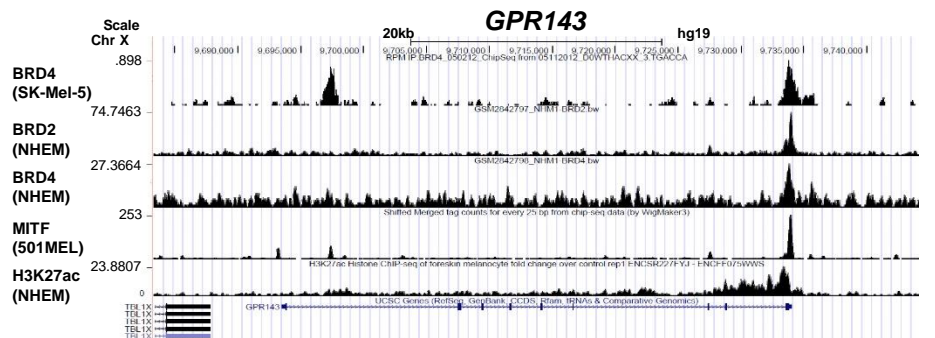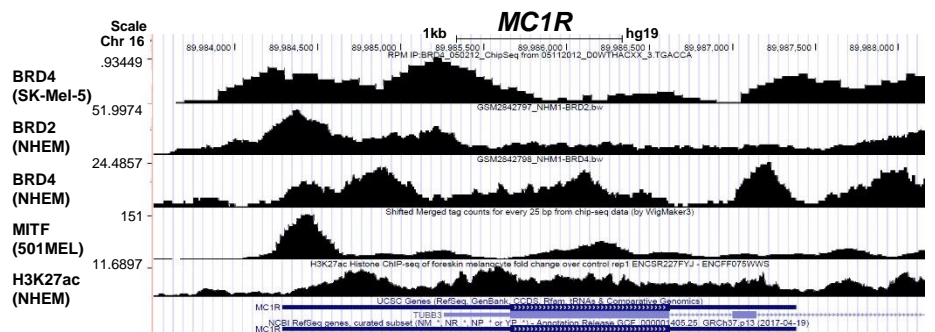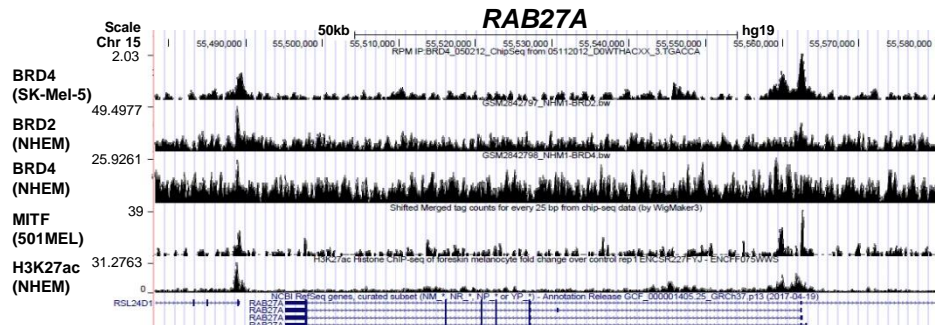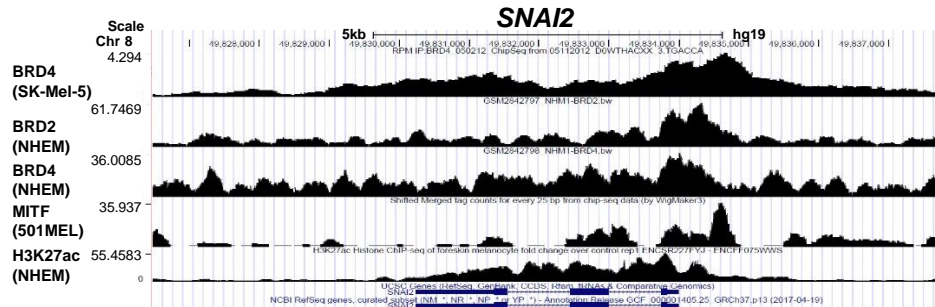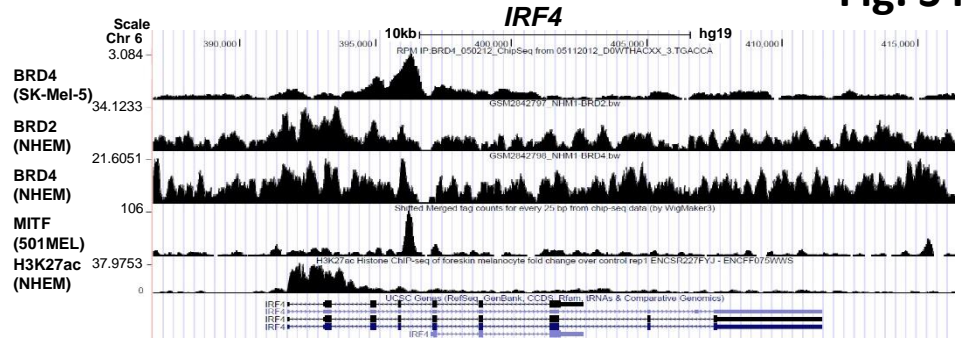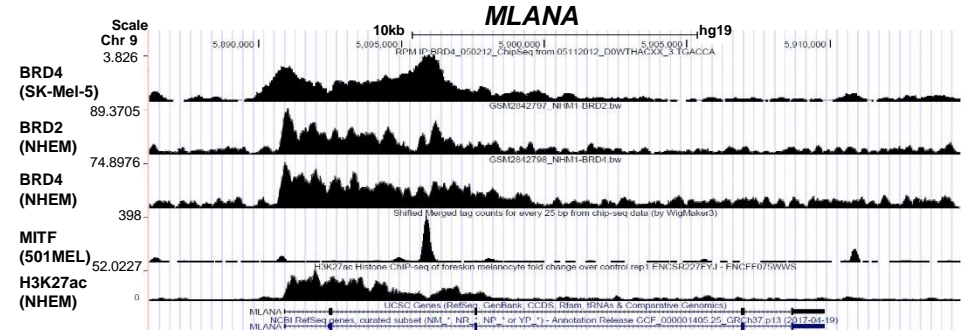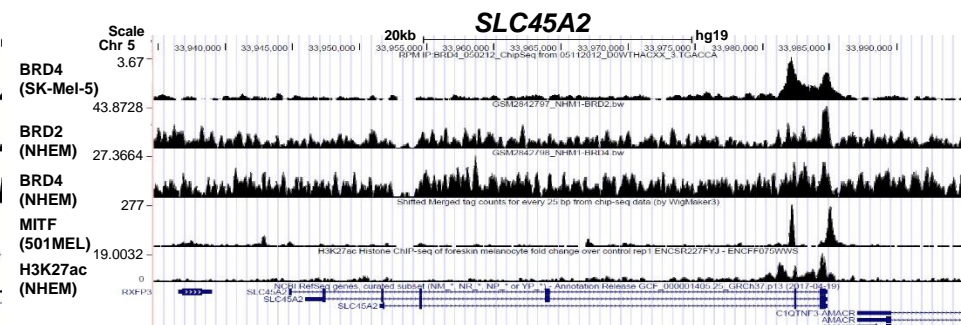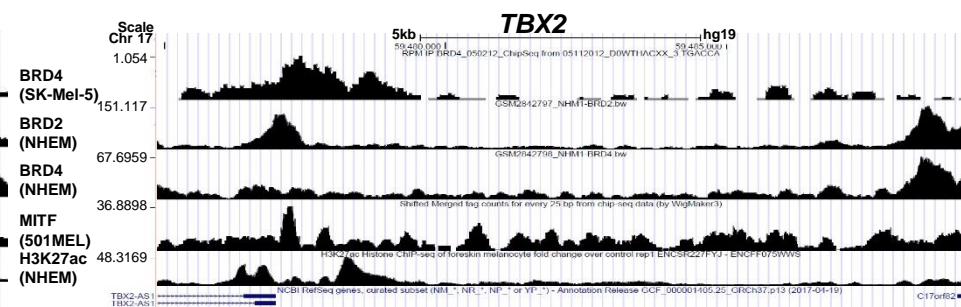

Supplement: Supplementary file 6 — Additional file 6: Fig. S4. Publicly available ChIP-seq data in normal human melanocytes, SK-MEL-5 melanoma cells (GSM1968282), 501 melanoma cells (GSM1517751), and neonatal human epidermal melanocytes (NHEM) (GSM2842798) with the indicated antibodies at the MITF target genes that are down-regulated by (+)JQ1 treatment. [file 13072_2020_333_MOESM6_ESM.pdf]

Fig. S5

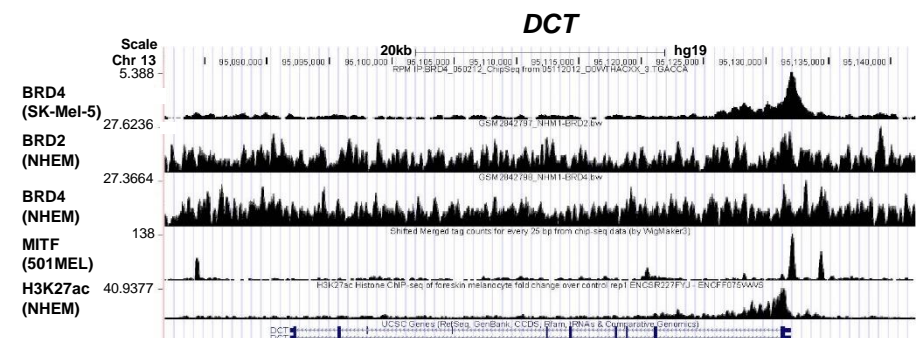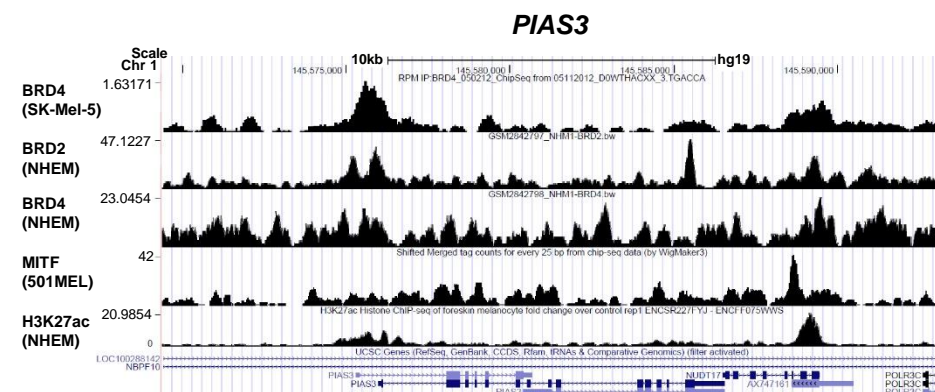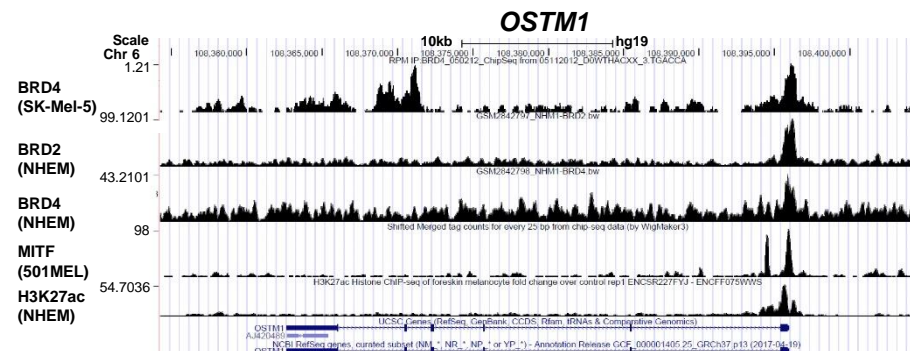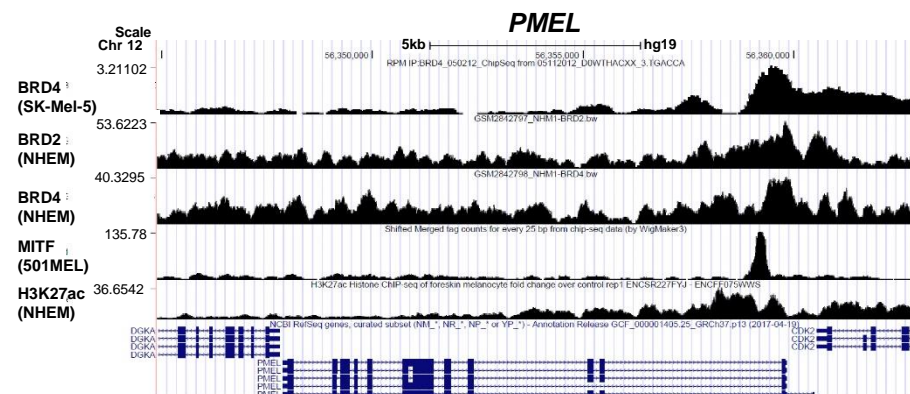

Supplement: Supplementary file 7 — Additional file 7: Fig. S5. Publicly available ChIP-seq data in normal human melanocytes, SK-MEL-5 melanoma cells (GSM1968282), 501 melanoma cells (GSM1517751), and neonatal human epidermal melanocytes (NHEM) (GSM2842798) with the indicated antibodies at the MITF target genes that are up-regulated by (+)JQ1 treatment. [file 13072_2020_333_MOESM7_ESM.pdf]
