# Supplementary material for: Bromodomain and extra-terminal domain (BET) proteins regulate melanocyte differentiation
Source: Epigenetics Chromatin. 2020 Mar 10;13:14. doi: 10.1186/s13072-020-00333-z (PMC7063807; doi:10.1186/s13072-020-00333-z)
Supplement: Supplementary file 3 — Additional file 3: Fig. S3. Melb-a cells were differentiated for 48 h in the presence of vehicle or 500nM +JQ1. RNA was isolated and subjected to RNA-seq. Differential gene expression ((+)JQ1 vs. Vehicle) was determined based on the average of three biological replicates (adj. p < 0.05). A. Volcano plot showing down-regulated gene expression (green) and up-regulated gene expression (red). B. Gene ontology analysis of up-regulated biological processes, cellular components, molecular function. C. Gene ontology analysis of down-regulated biological processes, cellular components, molecular function. D. Kegg Pathway analysis of up-regulated pathways. E. Kegg Pathway analysis of down-regulated pathways. [file 13072_2020_333_MOESM3_ESM.pdf]

Fig. S3

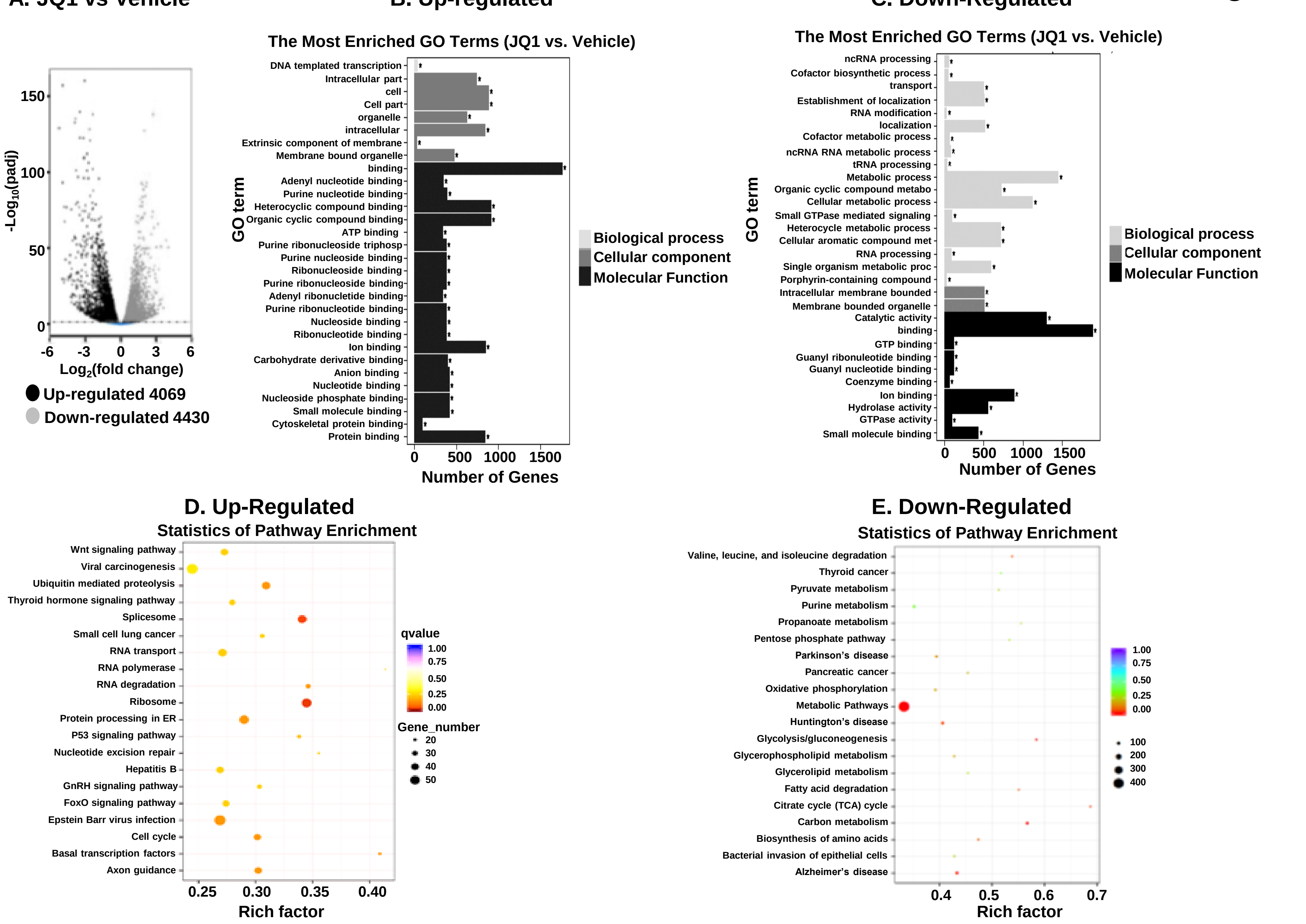
A. JQ1 vs Vehicle
-Log10(padj)
150
100
50
0
-6
-3
0
3
6
Log2(fold change)
Up-regulated 4069
Down-regulated 4430
B. Up-regulated
The Most Enriched GO Terms (JQ1 vs. Vehicle)
GO term
DNA templated transcription
Intracellular part
cell
Cell part
organelle
intracellular
Extrinsic component of membrane
Membrane bound organelle
binding
Adenyl nucleotide binding
Purine nucleotide binding
Heterocyclic compound binding
Organic cyclic compound binding
ATP binding
Purine ribonucleoside triphosp
Purine nucleoside binding
Ribonucleoside binding
Purine ribonucleoside binding
Adenyl ribonucletide binding
Purine ribonucleotide binding
Nucleoside binding
Ribonucleotide binding
Ion binding
Carbohydrate derivative binding
Anion binding
Nucleotide binding
Nucleoside phosphate binding
Small molecule binding
Cytoskeletal protein binding
Protein binding
0
500
1000
1500
Number of Genes
Biological process
Cellular component
Molecular Function
C. Down-Regulated
The Most Enriched GO Terms (JQ1 vs. Vehicle)
GO term
ncRNA processing
Cofactor biosynthetic process
transport
Establishment of localization
RNA modification
localization
Cofactor metabolic process
ncRNA RNA metabolic process
tRNA processing
Metabolic process
Organic cyclic compound metabo
Cellular metabolic process
Small GTPase mediated signaling
Heterocycle metabolic process
Cellular aromatic compound met
RNA processing
Single organism metabolic proc
Porphyrin-containing compound
Intracellular membrane bounded
Membrane bounded organelle
Catalytic activity
binding
GTP binding
Guanyl ribonuleotide binding
Guanyl nucleotide binding
Coenzyme binding
Ion binding
Hydrolase activity
GTPase activity
Small molecule binding
0
500
1000
1500
Number of Genes
Biological process
Cellular component
Molecular Function
D. Up-Regulated
Statistics of Pathway Enrichment
Wnt signaling pathway
Viral carcinogenesis
Ubiquitin mediated proteolysis
Thyroid hormone signaling pathway
Splicesome
Small cell lung cancer
RNA transport
RNA polymerase
RNA degradation
Ribosome
Protein processing in ER
P53 signaling pathway
Nucleotide excision repair
Hepatitis B
GnRH signaling pathway
FoxO signaling pathway
Epstein Barr virus infection
Cell cycle
Basal transcription factors
Axon guidance
0.25
0.30
0.35
0.40
Rich factor
qvalue
1.00
0.75
0.50
0.25
0.00
Gene_number
20
30
40
50
E. Down-Regulated
Statistics of Pathway Enrichment
Valine, leucine, and isoleucine degradation
Thyroid cancer
Pyruvate metabolism
Purine metabolism
Propanoate metabolism
Pentose phosphate pathway
Parkinson's disease
Pancreatic cancer
Oxidative phosphorylation
Metabolic Pathways
Huntington's disease
Glycolysis/gluconeogenesis
Glycerophospholipid metabolism
Glycerolipid metabolism
Fatty acid degradation
Citrate cycle (TCA) cycle
Carbon metabolism
Biosynthesis of amino acids
Bacterial invasion of epithelial cells
Alzheimer's disease
0.4
0.5
0.6
0.7
Rich factor
1.00
0.75
0.50
0.25
0.00
100
200
300
400
